# Psychostimulants for the Treatment of Comorbid Post-traumatic Stress Disorder (PTSD) in a Patient With Attention-Deficit/Hyperactivity Disorder (ADHD): A Case Report and Literature Summary

**DOI:** 10.7759/cureus.28199

**Published:** 2022-08-20

**Authors:** Claudette Barreto, Ana Vila Irigoyen, Olga Lopez, Leonard Gralnik

**Affiliations:** 1 Medicine, Florida International University, Herbert Wertheim College of Medicine, Miami, USA; 2 Psychiatry, Florida International University, Herbert Wertheim College of Medicine, Miami, USA

**Keywords:** post-traumatic stress disorder (ptsd), attention-deficit/hyperactivity disorder (adhd), psychostimulants, intrusive thoughts, schedule ii stimulants, stimulants use

## Abstract

We present a case of a 35-year-old female with an extensive history of attention-deficit/hyperactivity disorder (ADHD) who experienced a traumatic sexual and physical assault and later developed post-traumatic stress disorder (PTSD). The patient disclosed that her current medication (Vyvanse) used to treat ADHD has been the only treatment modality that has helped control her intrusive thoughts and nightmares. Decreased dopaminergic receptors are associated with developing PTSD and psychostimulants are known to have an effect of increasing Dopamine release. This case report shows promising results and potential off-label use of psychostimulants for the treatment of PTSD.

## Introduction

Post-traumatic stress disorder (PTSD) is diagnosed in patients who have experienced exposure to actual or threatened death, serious injury, or sexual violence. Following this traumatic event, they then experience intrusive symptoms associated with the traumatic event such as distressing memories or dreams, persistent avoidance of stimuli, negative alterations in cognitions and mood, and marked alterations in arousal and reactivity. The disturbance must have a duration of more than one month, must cause significant distress or impairment, and cannot be attributed to the physiological effects of a substance [1]. The lifetime prevalence of PTSD ranges from 6.1% to 9.2% with a one-year prevalence rate ranging from 3.5% to 4.7%. The overall prevalence for females is higher than the prevalence for males. Impairment from PTSD was explored using the Sheehan Disability Scale. There was an estimated 36.6% of adults with serious impairment, and 30.2% of adults suffering from PTSD experiencing mild impairment [2].

Attention-deficit/hyperactivity disorder (ADHD) is diagnosed in both children and adults who have persistent inattention and/or hyperactivity-impulsivity that interferes with daily life. For adults, five or more symptoms of inattention and/or hyperactivity-impulsivity must be present for at least six months. In addition, several of these symptoms had to be present before the age of 12 years and need to be displayed in two or more different settings. Symptoms must not be better explained by another mental disorder or medical condition [1]. The overall prevalence of ADHD in the United States among adults aged 18-44 years is 4.4%. This prevalence is higher for males and non-Hispanic white groups [3]. Pharmacotherapy for ADHD consists of stimulants including amphetamines and methylphenidate and non-stimulants including atomoxetine, clonidine, and guanfacine [4].

In 2010, three cases of treatment-resistant combat-related PTSD were described that were found to respond well to psychostimulant medications. Though the etiology of PTSD is multifactorial and not entirely understood, there is a correlation between decreased dopamine activity in the prefrontal cortex, basal ganglia system, and being more vulnerable to develop PTSD later. The psychostimulant effects of increased catecholamine release, specifically Dopamine shows potential for stimulants to be used as an off-label treatment for PTSD [5]. 

## Case presentation

The patient is a 35-year-old female with a history of ADHD and PTSD secondary to sexual assault. The patient initially presented in 2015 to establish psychiatric care at our center. At that time, she was treated for ADHD with Vyvanse 30 mg daily. She stated that her diagnosis of ADHD was established when she was a young child due to hyperactivity and problems with concentration and attention. She was previously treated with Ritalin in elementary school and had also been prescribed Adderall later in high school. However, she discontinued the medication due to gastrointestinal side effects. She had mentioned that the Vyvanse was working well for her but at that time, she was hoping to taper off given that she was interested in having a child. In 2019, the patient stated that the 30 mg of Vyvanse was not a sufficient dose to control her symptoms of inattentiveness and the dose was subsequently increased to 40 mg. The patient tolerated this dose well with no unwanted side effects. 

During a follow-up visit in the early half of 2020, the patient disclosed for the first time that she had been raped and sustained multiple physical injuries while jogging in 2008. She stated that since this occurred, she had been having symptoms of PTSD such as vivid flashbacks which had resolved when she began therapy with Vyvanse in 2009. She had been reluctant to disclose this information about the sexual assault in prior visits. In addition, she stated that a combined medical and therapeutic approach was helpful. In the latter half of 2020, the patient developed an upper respiratory infection which resulted in around three days of missed Vyvanse doses which she reported caused her to lose her focus. The intrusive thoughts associated with the assault had then returned and this also compromised her sleep. She reported that the maximum number of subsequent days she is able to miss her Vyvanse is two days but more than this causes her mental status to worsen, with a significant exacerbation of distressing PTSD symptoms. The patient also mentioned that when the Vyvanse dosage is lowered to less than 40 mg, her quality of sleep begins to worsen due to intrusive thoughts and she also finds it harder to sit still. At the beginning of 2021, when she was continuing psychotherapy but had briefly discontinued that Vyvanse, she had been attempting to conceive and had a miscarriage at week eight of her pregnancy. At the beginning of 2022, the patient and her spouse decided to opt for adoption; the patient believes this is the best option for her and will also allow her to continue her treatment with Vyvanse to both control her ADHD and her PTSD. She believes that Vyvanse 40 mg is the minimal effective dose for her at this time. 

Social history

The patient works as an attorney, and her job is stressful at times. She has been married since 2011 and she described her relationship with her husband as healthy and supportive. They have been wanting to have children but have been unsuccessful and have opted for adoption and are currently going through this process. The patient maintains a healthy lifestyle and previously ran track and field in college, and continues to run on a weekly basis. She denies using tobacco products or illicit drugs of any sort but does admit to using alcohol on a social basis on occasion.

Medical and family history

The patient is allergic to both amoxicillin and penicillin. She has previously had a tonsillectomy and surgery to the right knee following her traumatic assault. Family history is significant for her father with ADHD. There is no other known family psychiatric or drug use history.

## Discussion

Given the lifetime worldwide prevalence of PTSD in adults can be upwards of 9%, with a greater prevalence reported in females than males; addressing this psychiatric disorder with novel treatments and therapy modalities is essential [2]. There are various options available for treating PTSD which typically consist of a combination of pharmacological and psychotherapeutic approaches, however, the possibility of utilizing stimulants to treat PTSD still remains heavily understudied. Daly was among the first to report a case of a patient suffering from PTSD who was treated with diethylpropion HCl. This stimulant is a phenethylamine which although structurally different from amphetamine results in similar effects [6]. 

More recently, Houlihan published a case series outlining the use of psychostimulants in three cases of veterans in the United States. The results of this study showed that psychostimulant treatment demonstrated improvement in the overall symptoms of PTSD for the patients in this study while their previous gold standard therapies failed [5]. This case series also elucidated a potential mechanism for the efficacy of psychostimulants in the treatment of PTSD, which may have to do with the interplay between the catecholaminergic and subsequent dopaminergic neural axes [7,8]. This largely may have to do with the role that stimulants play with their indirect increase of dopamine in regions, such as the nucleus accumbens and the prefrontal cortex [9,10]. Other studies such as those by Blum et al. support this theory and hypothesize that psychostimulants may also lead to increased proliferation of dopaminergic receptors further leading to an increase in the levels of dopamine activity in similar regions [11]. 

On the contrary, a more recent cohort study Crum-Cianflone et al. examined the association between the use of psychostimulants and the development of PTSD in active duty U.S. military members. They found a greater incidence of PTSD in those who had a history of psychostimulant use even after completing a sub-group analysis controlling for confounding variables [12]. 

Another concern regarding the potential future usage of psychostimulants to treat PTSD is the potential for the development of substance use disorder in such individuals. Significant comorbidity for patients with PTSD is substance-use disorder. Despite this concern, psychostimulants have a lower addictive potential than illicit stimulants [5]. Patients successfully treated with psychostimulants may minimize self-medication with illicit stimulants.

## Conclusions

Given the lack of research regarding this topic in the current body of literature, this case report serves to highlight the need for ongoing research regarding the use of psychostimulants to treat PTSD. There is some evidence for this therapeutic approach, however, there is a need for more robust trials regarding this topic. Our study described the successful use of Vyvanse to treat PTSD (along with ADHD) in a single patient at our institution. We believe that further case series or randomized controlled trials evaluating the use of psychostimulants compared to the gold standard treatment for PTSD will elucidate useful and potentially life-saving modalities in the treatment of this psychiatric disorder.

It should be re-emphasized that this patient was initially being treated for ADHD and her care team was not aware of her PTSD symptoms until she revealed them to us much later in the course of her treatment. Over the course of this patient’s treatment, it became very clear that psychostimulant treatment led to a consistent and robust improvement in her PTSD symptoms, and a clear relapse in PTSD symptoms whenever the psychostimulant was discontinued, even for a few days. She concurrently benefited from the psychostimulant with good control of her ADHD symptoms, but her response to psychostimulant treatment of PTSD seemed separate and distinct from the treatment of her comorbid ADHD. This was a consistent effect over at least two or three episodes where her PTSD symptoms recurred when she stopped the stimulants, and these episodes were well-controlled rapidly upon restarting the stimulants. The patient reported that when her inattentiveness recurred upon discontinuation of the stimulant, her mind would wander and then the intrusive, distressing PTSD-related memories would be exacerbated, causing her great distress. There is a need for further studies, but we suggest that the treatment of PTSD with psychostimulants such as Vyvanse should be considered in some patients, particularly those with a well-established comorbid diagnosis of ADHD.
